# Effects of administration of basic fibroblast growth factor on hypoxic fractions in xenografted DLD-2 human tumours: time dependence.

**DOI:** 10.1038/bjc.1993.418

**Published:** 1993-10

**Authors:** J. T. Leith, S. Michelson

**Affiliations:** Department of Radiation Medicine, Brown University School of Medicine, Providence, Rhode Island 02912.

## Abstract

A previous publication (Leith et al., 1992) showed that administration of basic fibroblast growth factor (FGF-2, 0.25 mg kg-1, q.i.d. x 7) to mice bearing xenografted DLD-2 human colon cancers would increase treated tumour growth rates as compared to control neoplasms. Additionally, at the end of the 7 day treatment period, clonogenic excision assays showed that the percentage of hypoxic cells in tumours from mice receiving FGF-2 administration was significantly decreased as compared to control neoplasms (from about 42 to about 19%). The present study was undertaken to better define the kinetics of changes in hypoxic percentages as a function of tumour volume and FGF-2 treatment. In sham-injected control tumours, the hypoxic percentage increased from about 14% at day 15 postimplantation, (i.e. when sham- or FGF-2 injections were begun) to about 42% by day 22, and to about 75% at 29 days postimplantation (respective average volumes 220, 910, and 2810 mm3). In contrast, the hypoxic percentages in mice treated with FGF-2 remained at the levels seen in control mice on day 15, not only throughout the 7 day FGF-2 treatment schedule, but for at least 1 week after the cessation of growth factor administration. The hypoxic percentage was 16% on day 29 postimplantation, even though average tumour volumes were about 4325 mm3. These data show that the effect of FGF-2 administration on tumour growth rate and hypoxic percentages in xenografted DLD-2 neoplasms is rapid, and continues for some period of time even after administration is ended. Studies of tumour perfusion with injected 86RbCl at equivalent tumour volumes of about 1800 mm3 indicated that the percentage of cardiac output to FGF-2 treated tumours was 33% greater than in sham-injected control neoplasms.


Br. J. Cancer (1993), **68**, 727-731 © Macmillan Press Ltd., 1993

### Effects of administration of basic fibroblast growth factor on hypoxic fractions in xenografted DLD-2 human tumours: Time dependence

J.T. Leith[1] & Seth Michelson[2]

[1]*Radiation Research Laboratories, Department of Radiation Medicine, Brown University School of Medicine, Providence, Rhode Island 02912, USA, and* [2]*Biomathematics and Research Data Management Group, Syntex Corporation, Palo Alto, California 94303, USA.*

**Summary** A previous publication (Leith *et al.*, 1992) showed that administration of basic fibroblast growth factor (FGF-2, 0.25 mg $kg^{-1}$, q.i.d. × 7) to mice bearing xenografted DLD-2 human colon cancers would increase treated tumour growth rates as compared to control neoplasms. Additionally, at the end of the 7 day treatment period, clonogenic excision assays showed that the percentage of hypoxic cells in tumours from mice receiving FGF-2 administration was significantly decreased as compared to control neoplasms (from about 42 to about 19%). The present study was undertaken to better define the kinetics of changes in hypoxic percentages as a function of tumour volume and FGF-2 treatment. In sham-injected control tumours, the hypoxic percentage increased from about 14% at day 15 postimplantation, (i.e. when sham- or FGF-2 injections were begun) to about 42% by day 22, and to about 75% at 29 days postimplantation (respective average volumes 220, 910, and 2810 $mm^3$). In contrast, the hypoxic percentages in mice treated with FGF-2 remained at the levels seen in control mice on day 15, not only throughout the 7 day FGF-2 treatment schedule, but for at least 1 week after the cessation of growth factor administration. The hypoxic percentage was 16% on day 29 postimplantation, even though everage tumour volumes were about 4325 $mm^3$. These data show that the effect of FGF-2 administration on tumour growth rate and hypoxic percentages in xenografted DLD-2 neoplasms is rapid, and continues for some period of time even after administration is ended. Studies of tumour perfusion with injected $^{86}RbCl$ at equivalent tumour volumes of about 1800 $mm^3$ indicated that the percentage of cardiac output to FGF-2 treated tumours was 33% greater than in sham-injected control neoplasms.

We recently reported in this journal that administration of basic fibroblast growth factor (FGF-2; q.i.d. × 7; 0.25 mg $kg^{-1}$) caused a significant increase in the growth rates of xenografted DLD-2 human colon cancers, and concomitantly decreased the percentage of hypoxia within these neoplasms (Leith *et al.*, 1992). In that study, hypoxia levels were determined 1 day after the end of a 7 day period of FGF-2 administration, at a time when the volumes of control (given sham-injections with Hank's balanced salt solution) or FGF-2 treated tumours had increased from 211 $mm^3$ to 992 and 1751 $mm^3$ respectively. Although the FGF-2 treated tumours were about 1.8 times the volume of controls at the time of assay, the percentage of hypoxia within these neoplasm was 19.1% (13.5–26.9; 95% confidence limits) whereas the percentage in control neoplasms was 42.2% (34.2–52.1). In essence, increases in tumour volume can be uncoupled from changes in hypoxia by FGF-2 administration. In these studies however, the effects of the nonequivalent tumour volumes on interpretation of steady-state levels of hypoxia as a function of volume were not explicitly studied.

Because of our lack of knowledge of how steady-state levels of hypoxia within unperturbed DLD-2 tumours might change as a function of volume, we performed further experiments to define these kinetics. Additionally, we examined hypoxia levels in FGF-2 treated DLD-2 tumours at several times during and after the administration of growth factor.

### Materials and methods

### *Cell line*

The biological characteristics of the DLD-2 cell line have been previously described (Leith *et al.*, 1992). For these experiments, stock cells stored in liquid nitrogen were grown in RPMI-1640 medium containing 10% foetal bovine serum (FBS), 1% sodium bicarbonate, 1% anti-PPLO reagent, 1% 4-(2-hydroxyethyl)-1-piperazineethanesulfonic acid buffer, and 0.04% gentamicin (all reagents from the Grand Island Biological Co., Grand, Island, NY).

### *Mice and production of xenografted tumours*

Young adult male mice were obtained from the Charles River Breeding Laboratories, North Wilmington, MA. Mice were housed, five per large cage, with dust covers, in a dedicated room in the Brown University Animal Care Facilities in a laminar flow hood (Thoren Industries, King of Prussia, PA). Mice were quarantined for 1 week, and were ear tagged for identification. To produce tumours (one tumour per animal), DLD-2 cells were trypsinised (0.05% trypsin, 0.54 mM EDTA) from exponentially growing cultures, and resuspended as single cells in Hank's basic salt solution (HBSS) at a concentration of $5 \times 10^7$ cells $ml^{-1}$. Nought point two ml of the cell suspension was injected into the right flank regions of the mice.

### *Volumetric procedures*

Tumours were measured in two orthogonal diameters and volumes ($mm^3$) were calculated using the formula for a prolate ellipsoid [V ($mm^3$) = L × $W^2$/2] where L and W are respectively the major and minor diameters (Leith *et al.*, 1992). All measurements were made by a single individual. After injection, tumours were monitored until average sizes of 220 $mm^3$ were reached, at which time animals were randomly assigned to control or FGF-2 groups. The tumour sizes in this work are comparable to those used in our previous study on the effects of FGF-2 (Leigh *et al.*, 1992).

### *Treatment of nude mice bearing DLD-2 xenografts with FGF-2*

Recombinant human FGF-2 (purity > 98%) obtained from Bachem Bioscience, Inc., Philadelphia, PA was used. FGF-2 was reconstituted from lyophilised powder using HBSS, and was stored for short periods of time at − 20°C in HBSS at a concentration of 1 μg $\mu l^{-1}$. The endotoxin level as noted by the company was less than 0.1 ng $\mu g^{-1}$ of FGF-2. FGF-2 was

Correspondence: J.T. Leith, Radiation Research Laboratories, Box G, Rm. B-004, Brown University School of Medicine, Providence, RI 02912, USA.
Received 20 November 1992; and in revised form 7 June 1993.

administered i.p. at a dose of 0.25 mg $kg^{-1}$ $day^{-1}$ for a period of 7 days, in keeping with our previously published protocol (Leith *et al.*, 1992). Sham injections were done using HBSS.

### *Determination of hypoxic fractions of xenografted DLD-2 tumours*

Tumours were irradiated in either air-breathing, unanaesthetised mice or mice that had been asphyxiated by a 10 min exposure to nitrogen gas prior to irradition (Leith *et al.*, 1992). For irradiations, mice were briefly anesthaetised with Metofane (methoxyflurane; Pitman-Moore, Inc., Washington Crossing, NJ) and restrained on a lucite irradiation platform. Animals were allowed to fully recover from the anaesthesia, and were then irradiated at room temperature using a Philips 250 kVp X-ray machine (Philips X-Ray, New Bedford, MA), operated at 250 kV and 15 mA. Exposure doses were measured using a Victoreen R-meter (Victoreen Co., Cleveland, OH), and absorbed doses were calculated using appropriate temperature, pressure, and Roentgen to Gy conversion factors. The absorbed dose rate was about 1 Gy $min^{-1}$.

For determinations of clonogenic cell survival by excision assay, we delivered graded doses of 0-25 Gy to oxic and hypoxic tumours in either control or FGF-2 treated mice. Immediately after irradiations, neoplasms were excised under sterile conditions, quartered, placed into ice-cold HBSS, and weighed. Then the pieces were minced using opposed scalpel blades into approximately 1 $mm^3$ fragments, and placed into an enzyme cocktail containing 0.2% RNase free DNase (Sigma Chemical Co., St. Louis, MO), 0.25% collagenase (Boehringer Mannheim Biochemicals, Indianapolis, IN), and 0.25% neutral pronase (Calbiochem Corp., San Diego, CA) in RPMI-1640 medium without FBS. Tumour fragments were digested for 40 min at 37°C in stirred 250 ml trypsinising flasks. The digestate was filtered through an 80μM rectangular stainless steel mesh and pelleted (after addition of an equal volume of cold RPMI-1640 medium with FBS) at 1,000 r.p.m. for 10 min at 4°C. The pellet was resuspended in RPMI-1640 medium with FBS, tumour cells were counted using phase contrast microscopy, and appropriate numbers of cells were then seeded into 60 or 100 mm diameter plastic dishes (B-D Labware, Trenton, NJ) at several dilutions for enumeration of survival by colony formation. Heavily irradiated (30 Gy, $^{137}Cs$ gamma-rays; Model 68A irradiator, J.L. Sheperd Co., Glendale, CA) DLD-2 feeder cells were added to all dishes to keep a minimum cell number of $10^5$ cells/60 mm dish because the colony forming efficiency of DLD-2 cells is feeder cell dependent (Leith *et al.*, 1992). Colonies were allowed to develop at 37°C in a humidified incubator under an atmosphere of 5% $CO_2$ and 95% air for 10–14 days, after which time colonies were fixed and stained with 0.5% crystal violet in absolute methanol. To calculate hypoxic percentages at each dose point (5, 10, 15, 20, and 25 Gy) for oxic or hypoxic control of FGF-2 tumours, we first calculated the mean survival values from the 4–6 mice used in each determination of survival. The average survival values for oxic or hypoxic curves were then fit using linear regression analysis of log survival *vs* dose to determine the slopes of the curves and their 95% confidence limits (Goldstein, 1964). The regression equations were statistically compared to determine that the respective oxic and survival curves were parallel over the 5–25 Gy dose range (i.e., that the 95% confidence limits on estimations on the slopes of the curves overlapped). At each dose point (5–25 Gy), the hypoxic fraction (HF) was then calculated using the relationship

$$\text{Log (HF)} = \log (S_o) - \log (S_h)$$

where $S_o$ and $S_h$ represent that respective mean survival values of cells from oxic and hypoxic tumours respectively. Therefore, five estimations of the HF were obtained from each comparison for each condition (i.e. control tumours, FGF-2 treated tumours, size/time dependence). The hypoxia value cited for each condition is the log mean of these five estimations, with the 95% confidence limits given by the log s.e.m. determined for each log mean times the two-tailed *t*-value appropriate for the sample number with (N-2) degrees of freedom (Goldstein, 1964).

### *Estimates of tumour perfusion*

In these studies, 0.1 ml (25 μCi) of $^{86}$RbCl in 0.9% saline (specific activity 1–12 mCi $mg^{-1}$; Amersham Radiochemicals, Arlington Heights, IL) was injected into the tail veins of unanaesthetised tumour bearing mice (Sapirstein, 1958). The percentage uptake of $^{86}$RB corresponds to the distribution of cardiac output. Mice were sacrificed by cervical dislocation after 2 min, and tumours were immediately excised, blotted, and placed into pre-weighed counting tubes. The tails of the mcie were also removed and counted to insure that the residual activity at the site of injection was less than 10% that of the injected solution. The radioactivity of the samples was determined using a Beckman Model G-1000 gamma-counter (Beckman Instruments, Palo Alto, CA), and are expressed as percent of activity injected $g^{-1}$ wet weight of tumour. Tumours were dried at 100°C for 24 h to insure that the FGF-2 treatment had not altered the water content of neoplasms (Lin & Song, 1990). There were 12 mice in both the FGF-2 and sham-injected control groups.

In these perfusion studies, FGF-2 treated or control tumours were compared at equivalent volumes. FGF-2 treated tumours were studied 1 day after the end of the 7 day administration schedule (day 22 postimplantation) when average tumour volumes were 1748 $mm^3$ (s.e.m. 244.6 $mm^3$). HBSS injected tumours were allowed to grow to the same average volume (1882 $mm^3$, s.e.m. 220.1 $mm^3$) as FGF-2 treated neoplasms, which occurred on day 26 post-implantation.

### Results

As we noted in our previous work (Leith *et al.*, 1992), administration of FGF-2 at a rate of 0.25 mg $kg^{-1}$ $day^{-1}$ over the 7 day administration period did not produce altera-

**Figure 1** Volumetric growth of xenografted human DLD-2 tumours in nude mice. Data are shown for tumour growth in animals receiving i.p. injections (q.i.d. × 7) of basic fibroblast growth factor (bFGF) (●, 0.25 mg $kg^{-1}$ $day^{-1}$), or Hank's basic salt solution (○, control). The time during which HBSS or bFGF injections were given is indicated by the box. The vertial lines in the Figure indicate the times at which excision assays were performed for determinations of hypoxic percentages. The dashed line represents the predicted growth rate of tumours after cessation of bFGF administration if tumours were to immediately resume growth rates as seen in control neoplasms of similar size. s.e.m.s are not shown for purposes of clarity, but were typically ~ 15–20% of the mean tumour volume values.

tions of animal weight or indications of systemic toxicity. The $LD_{50/30}$ for FGF-2 in this protocol is about 0.6 mg $kg^{-1}$ $day^{-1}$.

In Figure 1, the volumetric growth curves for control or FGF-2 treated tumours as a function of time after transplantation are shown. Similar to what we have previously reported, FGF-2 administration results in an increased growth rate. Tumours reached average volumes of 100 $mm^3$ at about 11.5 days after injection. Treatment with HBSS or FGF-2 was started on day 15 post injection, when average tumour volumes were 222.4 $mm^3$, and daily treatments continued for 7 days (days 15–21). Over this time period, the volumes of control tumours increased to 808.6 $mm^3$ while the volumes of the FGF-2 treated tumours increased to 1309.8 $mm^3$. Therefore, the volumes of FGF-2 treated neoplasms were about 60% greater than that of controls when FGF-2 treatment was stopped. Volumetric measurements in control or FGF-2 treated mice were continued beyond the end of the FGF-2 treatment period (days 22, 25, and 29). In Figure 1, we have indicated by the dashed line the volumetric path that the FGF-2 tumours would have been predicted to follow if they reverted instantly to the growth rates seen in control neoplasms of the same size. While the experimental data lie above the dashed line suggesting that there may be long term effects of the previous FGF-2 administration on tumour growth, the sizes of the errors on the individual data points do not allow this to be stated with significance.

In Figure 2, we present the clonogenic x-ray survival curves for cells from control or FGF-2 treated tumours as a function of volume. As there were no situations in which parallelism was not demonstrated, this then allowed us to determine the hypoxic fraction. In panel a, data for control neoplasms are shown. As tumours grow larger, the estimated hypoxic fractions also increase. In panel b, the data from the FGF-2 treated mice are shown. In these clonogenic assay studies, we thought it necessary to perform survival curve determinations on both oxic and hypoxic tumour cells from control or FGF-2 treated tumours at all assay times (volumes), because FGF-2 can act as a radioprotective agent (Haimovitz-Friedman *et al*., 1991). If radioprotection were to occur during these experiments, the result might have been to alter cell survival as determined from these clonogenic excision assays, and shift the relative vertical positions of the oxic and hypoxic survival curves from which hypoxic fractions were determined.

The cell yields from control of FGF-2 treated DLD-2 tumours were not significantly different at early times during the excision assays. There was however, a decrease in cell yields seen in the control tumours at the larger sizes (i.e., at 30 days posttransplantation). These data are summarised in Table I.

In Figure 3, we show a plot of the hypoxic percentages seen in the control and FGF-2 treated tumours as a function of volume. Several points are of interest. First, the hypoxic

**Figure 2** Survival of DLD-2 cells from solid tumours after graded dose x-irradiation. In panel **a**, survival is shown for cells from control tumours excised from air-breathing, unanaesthetised mice at 15 (○), 17 (●), 19 (△), 22 (▲), 25 (□), or 29 (■) days postimplantation. Each curve has been fit using a best-fit, least square linear regression of (log) percent hypoxia *vs* dose. Because the survival responses of cells taken from completely hypoxic tumours did not change as a function of time postimplantation, these data are represented by a single curve (◇). In panel **b**, similar data are shown for hypoxic or oxic cells excised from tumours given daily i.p. doses (q.i.d. × 7) of basic fibroblast growth factor (bFGF, 0.25 mg $kg^{-1}$, $day^{-1}$, days 15–21 postimplantation) (symbols: day 15 = ○ (repeated from panel **a**); day 17 = ●; day 19 = △; day 22 = ▲, day 25 = □; day 29 = ■). As there were no significant changes in the survival of either oxic or hypoxic cells from animals treated with bFGF as a function of time, a single line has been fit to these data, as indicated by the solid lines. For purposes of comparison, the dashed lines in panel **b** represent the survival of oxic or hypoxic control (non-bFGF treated) tumour cells (15 days postimplantation) as shown in panel **a**. There were 4–6 determinations per dose point for all conditions shown in panels **a** and **b**. There was no dependence on the s.e.m.s of the mean survival points shown in panels **a** and **b** on tumour volume at a given dose. s.e.m.s as a log percentage of the log mean survival values did however increase from 4.4% to 5.8, 15.7, 34.0, and 42.7% at doses of 5, 10, 15, 20, and 25 Gy respectively.

**Table I** Cell yields (CYs) and colony forming efficiencies (CFEs) of control or FGF-2 treated human colon tumour xenografts as a function of time after implantation

| | *Days after implantation* | | | | | |
|---|---|---|---|---|---|---|
| | *15* | *17* | *19* | *22* | *25* | *29* |
| Controls | | | | | | |
| CY[a] | 4.62 (0.33) | 5.09 (0.41) | 3.98 (0.29) | 4.43 (0.33) | 3.72 (0.22) | 2.76 (0.40) |
| CFE[b] | 6.01 (0.20) | 4.87 (0.38) | 6.10 (0.71) | 7.32 (0.37) | 3.51 (0.29) | 3.02 (0.68) |
| FGF-2[c] | | | | | | |
| CY | 6.01 (0.51) | 5.32 (0.19) | 4.76 (0.34) | 3.50 (0.50) | 4.19 (0.36) | 3.48 (0.29) |
| CFE | 5.30 (0.91) | 6.99 (0.38) | 4.81 (0.89) | 7.15 (1.02) | 4.00 (0.54) | 4.26 (0.55) |

[a]Cells $mg^{-1} \times 10^4$: mean and s.e.m. [b]Colony forming efficiency: mean and s.e.m. [c]FGF-2 administered daily from days 15 to 21 at a dose of $0.25\,mg\,kg^{-1}$.

**Figure 3** Changes in the percentage of hypoxia within tumours from either control (○) or basic fibroblast growth factor (●) treated mice as a function of tumour volume. The dashed line represents the predicted increase in intratumour hypoxia after cessation of bFGF administration if the response was similar to that seen in control neoplasms. The error bars are means and 95% confidence limits.

percentages in the FGF-2 treated mice remain constant at the value seen at the start of treatment (i.e. about 14%) in tumours with volumes of about $220\,mm^3$. Second, the effect is present even within 2 days of FGF-2 treatment. Third, even after FGF-2 administration is ended, the hypoxic percentage remains unchanged over the next 7 days (animals were sacrificed at this point as tumour volumes were becoming large). In the Figure, we have indicated by the dashed line what might have been observed if the hypoxic percentage in the tumours of the FGF-2 treated mice immediately began to increase at a rate similar to that seen in the control mice at the end of treatment. The dashed line predicts that the hypoxic percentage would have increased to about 30% in that 7 day period, rather than remain at the observed level of 19%.

These results also show that FGF-2 has not altered the positions of the hypoxic survival curves determined from control or FGF-2 treated tumours as a function of tumour size or treatment time, and suggest that FGF-2 is not acting as a radioprotective agent.

The uptake of $^{86}Rb$ in the FGF-2 or sham-injected tumours (%/g wet weight) was respectively 5.42 (0.75) and 4.09 (0.51) (values in parentheses are the 95% confidence limits), a difference which is significant at the 5% level of confidence (Goldstein, 1964). No significant change in tumour water content was caused by the FGF-2 injections as indicated by comparison of the weights of the 100°C dried samples.

### Discussion

The primary results of this study are, first, that hypoxic fractions increase significantly with volume in unperturbed DLD-2 neoplasms. This result is consistent with findings in other model tumour systems as summarised by Moulder & Rockwell (1984), and Rockwell & Moulder (1990). Second, FGF-2 administration prevents the steady increase in the percentage of hypoxia seen in DLD-2 control neoplasms of increasing size. Hypoxic percentages in FGF-2 treated DLD-2 human colon cancers remain at levels of 14–18%, while the levels in control neoplasms increase from 14 to about 42% over the same time period. This result is obtained even in the face of the fact that the FGF-2 treated neoplasms actually have larger volumes at the end of growth factor administration than do controls. Third, this inhibitory effect on hypoxia expression continues for at least 1 week after cessation of FGF-2 administration.

Relevant to the above, Gross *et al.* (1993), who have also studied the effects of exogenously administered FGF-2 on DLD-2 tumour growth, showed that DLD-2 cells lack high affinity receptors for FGF-2, and produce low levels of immunoreactive FGF-2 (about $3\,ng\,10^{-6}$ cells). Autoradiographic sections prepared from DLD-2 tumours in animals given [$^{125}I$]rHu-FGF-2 showed that the binding was to endothelial cells, not to parenchymal tumour cells. Gross *et al.* (1993) therefore explain the observed increases in the mass of FGF-2 treated DLD-2 tumours as a result of increased angio-genesis/mitogenesis of tumour endothelial cells. Although Gross *et al.* (1993) did not examine the binding of FGF-2 to endothelial cells in conjunction with time dependent changes in endothelial cell proliferation, Gospodarowicz *et al.* (1976) have shown that the proliferative response of cultured endothelial cells to increased levels of FGF-2 is essentially instantaneous. This would be consistent with the rapid increase in tumour volume, and inhibition of increases in intratumour hypoxia. Our finding that hypoxic percentages remain low for some time after cessation of FGF-2 administration would be consistent with a sustained FGF-2 induced increase in overall vascular capability, which may be due to FGF-2 binding by the tumour extracellular matrix during administration (e.g. heparan sulfate proteoglycans), with subsequent gradual release of bound FGF-2 by heparinase and other proteolytic enzymes (Flaumenhaft *et al.*, 1989, 1990; Folkman *et al.*, 1988; Vlodavsky *et al.*, 1991). It is fortuitous that, in our choice of DLD-2 neoplasms to study FGF-2 related changes in intratumour hypoxia, DLD-2 cells contain low levels of FGF-2. Consequently, the background paracrine FGF-2 signal from tumour cells (D'Amore, 1990) to endothelial cells would be low, producing a situation where demonstration of a tumour response to exogenously administered FGF-2 would be optimal. Conversely, the possibility arises that, for tumours in which parenchymal cells produce high levels of FGF-2, the vasculature might already be supplied with high levels of endogenous FGF-2, and therefore exogenous administration of FGF-2 would have no effect. Support for this is given by Gross *et al.* (1993), who found that administration of FGF-2 to rat C6 solid tumours had no effect on tumour size. However, C6 cells produce about 630% more immunoreactive FGF-2 per cell than do

DLD-2 cells. These results also raise the possibility that absolute levels of FGF-2 production by various tumour cells might be correlated, probably in an inverse manner, to steady-state levels of hypoxia. The results presented herein on the 33% increase in tumour perfusion as measured by $^{86}$RbCl uptake, taken together with the studies of Gross *et al.* (1993) identify the tumour vasculature as the target for FGF-2 induced changes in growth and hypoxia in this model tumour system. Still, as noted by Lin and Song (1990), our $^{86}$Rb measurements only indicate the percentage of cardiac output distributed to the tumour, and may not necessarily indicate a real change in tumour blood flow in ml $min^{-1}$. Additional studies on blood flow (e.g. with laser Doppler flowmetry) would be useful.

Our results suggest that administration of growth factors such as FGF-2 during the course of fractionated radiotherapy might serve to increase the curability of certain solid tumours if hypoxia is a major problem in curability (e.g. Gatenby *et al.*, 1988). Several other factors should be considered in this regard however. For example, Haimovitz-Friedman *et al.* (1991) have shown that FGF-2 acts as a radiation protector for bovine endothelial cells *in vitro*, increasing cell survival by increasing the recovery from radiation damage. While these results were obtained on normal tissue, one must consider whether tumour cells would be similarly protected. *In vitro*, addition of FGF-2 (up to 0.2 $\mu g\ ml^{-1}$) did not alter the shape of the graded dose x-ray survival curve for DLD-2 cells in either immediate or delayed plating experiments (J. Leigh, unpublished data, 1992). This lack of modification of radiation survival by FGF-2 is consistent with the observation of Gross *et al.* (1993) that DLD-2 cells lack high affinity receptors for FGF-2. Also, Rofstad (1992) has shown that, if reoxygenation is rapid and extensive during fractionated radiotherapy, overall tumour response may not be significantly influenced by hypoxia *per se*. Rofstad suggests that intrinsic radiation sensitivity and the rate of repopulation between radiation fractions may be the main factors governing overall radioresponsiveness. Given that our studies show that FGF-2 significantly alters tumour growth rates, comparative *in vivo* studies on FGF-2 effects on repopulation rates in tumours with and without high affinity receptors for FGF-2 would be of interest.

This work was supported by Grants from the National Cancer Institute of the National Institutes of Health (CA 50350-04 and 5 P30 CA 13943).

### References

D'AMORE, P.A. (1990). Modes of FGF release *in vivo* and *in vitro*. *Cancer & Metastasis Reviews*, **9**, 227–238.

FLAUMENHAFT, R., MOSCATELLI, D., SAKSELA, O. & RIFKIN, D.B. (1989). Role of extracellular matrix in the action of basic fibroblast growth factor: matrix as a source of growth factor for long-term stimulation of plasminogen activator production and DNA synthesis. *J. Cell Physiol.*, **140**, 75–81.

FLAUMENHAFT, R., MOSCATELLI, D. & RIFKIN, D.B. (1990). Heparin and heparin sulfate increase the radius of diffusion and action of basic fibroblast growth factor. *J. Cell Biol.*, **111**, 1651–1659.

FOLKMAN, J., KLAGSBRUN, M., SASSE, J., WADZINSKI, M., INGBER, D. & VLODAVSKY, I. (1988). A heparin binding angiogenic protein – basic fibroblast growth factor – is stored within basement membrane. *Am. J. Pathol.*, **130**, 115–120.

GATENBY, R.A., KESSLER, H.B., ROSENBLUM, J.S., COIA, L.R., MOLDOFSKY, P.J., HARTZ, W.H. & BRODER, G.J. (1988). Oxygen distribution in squamous cell carcinoma metastases and its relationship to outcome of radiation therapy. *Int. J. Radiat. Oncol. Biol. Phys.*, **14**, 831–838.

GOLDSTEIN, A. (1964). *Biostatistics: An Introductory Text*. p. 140–150. Acadmic Press: New York.

GOSPODAROWICZ, D., MORAN, J., BRAUN, D. & BIRDWELL, C. (1976). Clonal growth of bovine vascular endothelial cells: fibroblast growth factor as a survival agent. *Proc. Natl Acad. Sci. USA*, **11**, 4120–4124.

GROSS, J.L., HERBLIN, W.F., DUSAK, B.A., CZERNIAK, P., DIAMOND, M.D., SUN, T., EIDSVOOG, K., DEXTER, D.L. & YAYON, A. (1993). Effects of modulation of basic fibroblast growth factor on tumor growth *in vivo*. *J. Natl Cancer Inst.*, **85**, 121–131.

HAIMOVITZ-FRIEDMAN, A., VLODAVSKY, I., CHAUDHURI, A., WITTE, L. & FUKS, Z. (1991). Autocrine effects of fibroblast growth factor in repair of radiation damage in endothelial cells. *Cancer Res.*, **51**, 2552–2558.

LEITH, J.T., PAPA, G., QUARANTO, L. & MICHELSON, S. (1992). Modification of the volumetric growth responses and steady-state hypoxic fractions of xenografted DLD-2 human colon carcinomas by administration of basic fibroblast growth factor or suramin. *Br. J. Cancer*, **66**, 345–348.

LIN, J.-C. & SONG, C.W. (1990). Effects of hydralazine on the blood flow in RIF-1 tumors and normal tissues of mice. *Radiat. Res.*, **124**, 171–177.

MOULDER, J.E. & ROCKWELL, S. (1984). Hypoxic fractions of solid tumors: experimental techniques, methods of analysis, and a survey of existing data. *Int. J. Radiat. Oncol, Biol. Phys.*, **10**, 695–712.

ROCKWELL, S. & MOULDER, J.E. (1990). Hypoxic fractions of human tumors xenografted into nude mice: a review. *Int. J. Radiat. Oncol. Biol, Phys.*, **19**, 197–202.

ROFSTAD, E.K. (1992). Repopulation between radiation fractions in human melanoma xenografts. *Int. J. Radiat. Oncol. Biol. Phys.*, **23**, 63–68.

SAPIRSTEIN, L.A. (1958). Regional blood flow by fractional distribution of indicators. *Am. J. Physiol.*, **193**, 161–168.

VLODAVSKY, I., FUKS, Z., ISHAI-MICHAELI, R., BASHKIN, P., LEVI, E., KORNER, G., BAR-SHAVIT, R. & KLAGSBRUN, M. (1991). Extracellular matrix-resident basic fibroblast growth factor: implication for the control of angiogenesis. *J. Cell. Biochem.*, **45**, 167–176.


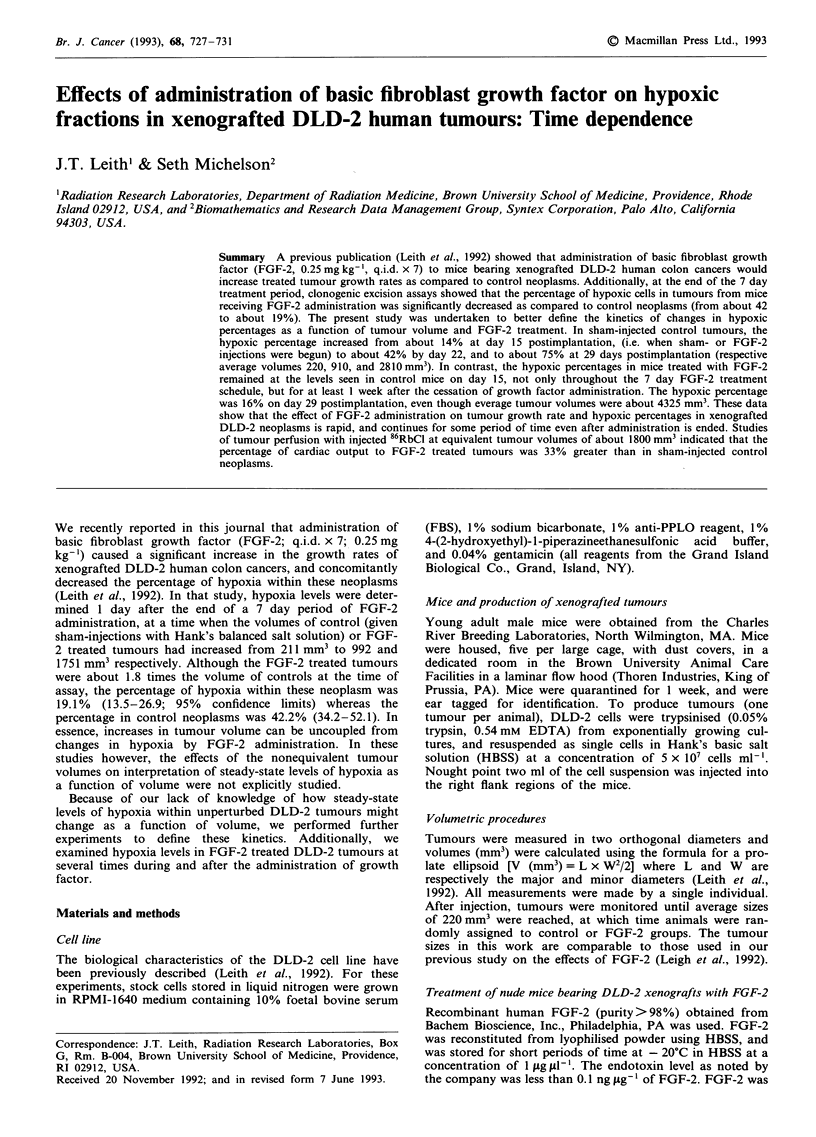

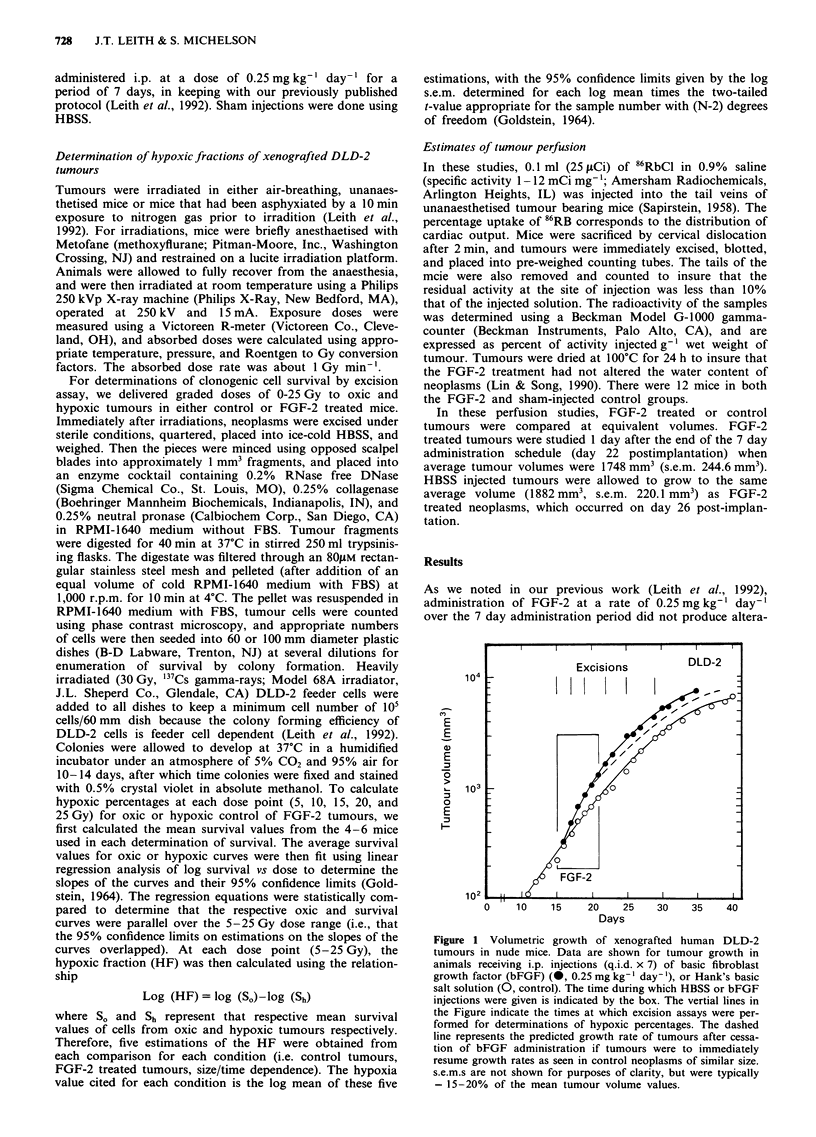

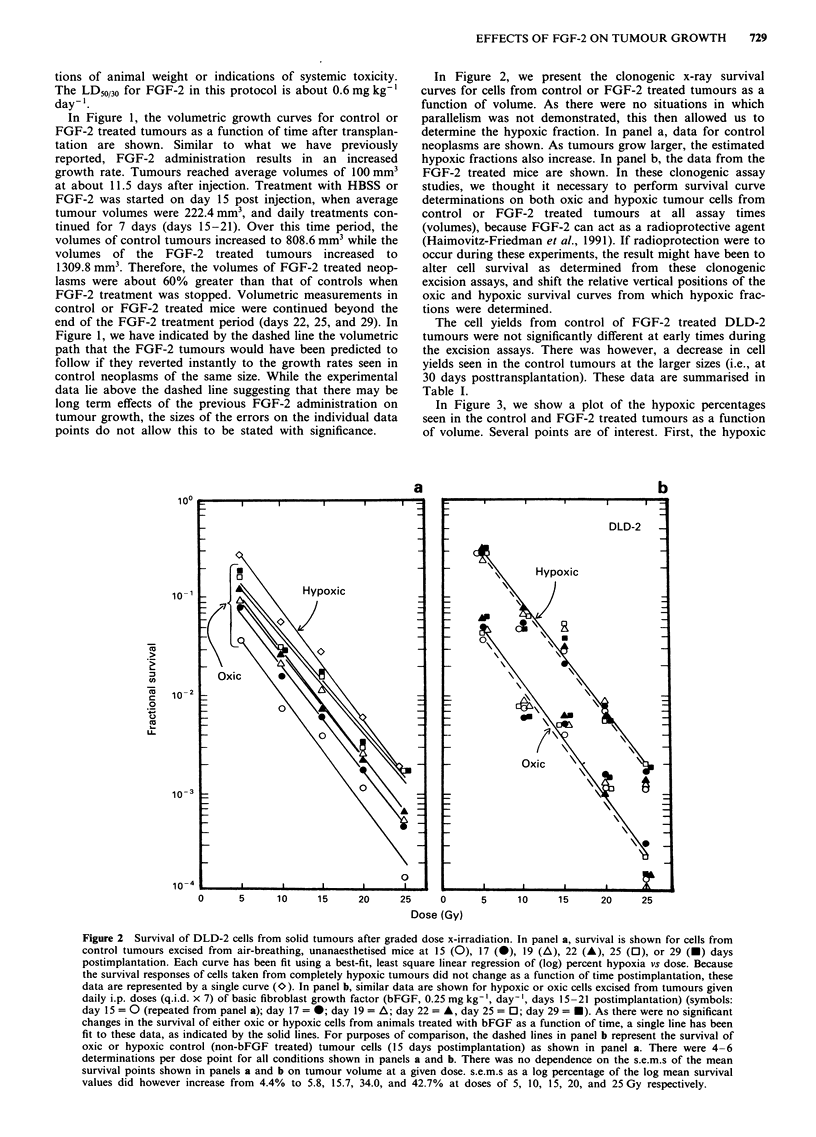

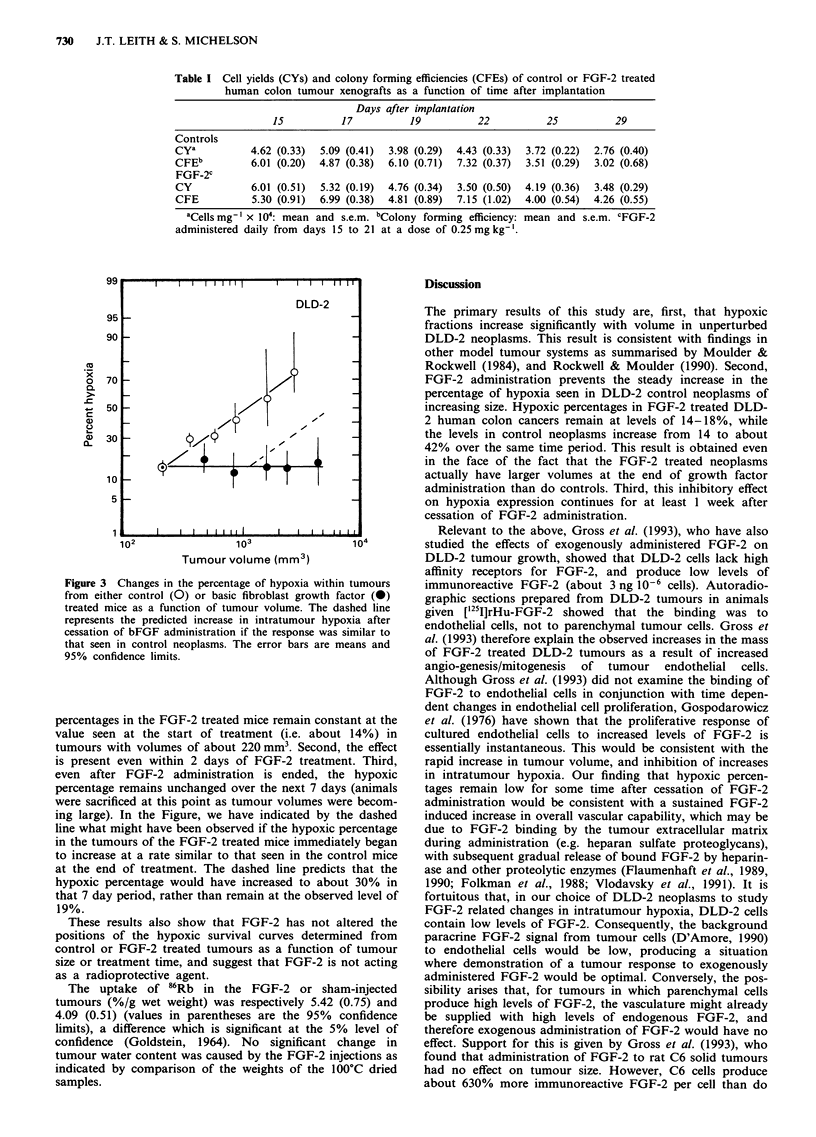

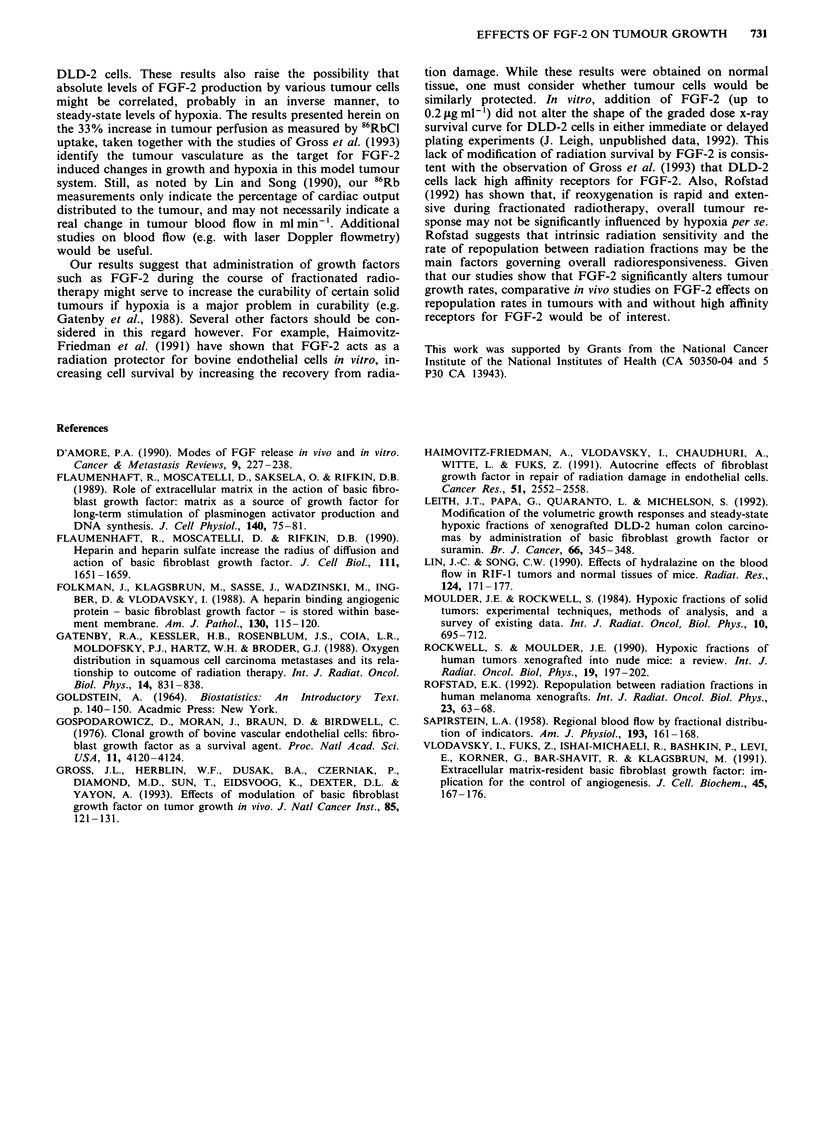


## References

[OCR_00607] D'Amore P. A. (1990). Modes of FGF release in vivo and in vitro.. Cancer Metastasis Rev.

[OCR_00618] Flaumenhaft R., Moscatelli D., Rifkin D. B. (1990). Heparin and heparan sulfate increase the radius of diffusion and action of basic fibroblast growth factor.. J Cell Biol.

[OCR_00611] Flaumenhaft R., Moscatelli D., Saksela O., Rifkin D. B. (1989). Role of extracellular matrix in the action of basic fibroblast growth factor: matrix as a source of growth factor for long-term stimulation of plasminogen activator production and DNA synthesis.. J Cell Physiol.

[OCR_00630] Gatenby R. A., Kessler H. B., Rosenblum J. S., Coia L. R., Moldofsky P. J., Hartz W. H., Broder G. J. (1988). Oxygen distribution in squamous cell carcinoma metastases and its relationship to outcome of radiation therapy.. Int J Radiat Oncol Biol Phys.

[OCR_00641] Gospodarowicz D., Moran J., Braun D., Birdwell C. (1976). Clonal growth of bovine vascular endothelial cells: fibroblast growth factor as a survival agent.. Proc Natl Acad Sci U S A.

[OCR_00647] Gross J. L., Herblin W. F., Dusak B. A., Czerniak P., Diamond M. D., Sun T., Eidsvoog K., Dexter D. L., Yayon A. (1993). Effects of modulation of basic fibroblast growth factor on tumor growth in vivo.. J Natl Cancer Inst.

[OCR_00654] Haimovitz-Friedman A., Vlodavsky I., Chaudhuri A., Witte L., Fuks Z. (1991). Autocrine effects of fibroblast growth factor in repair of radiation damage in endothelial cells.. Cancer Res.

[OCR_00660] Leith J. T., Papa G., Quaranto L., Michelson S. (1992). Modification of the volumetric growth responses and steady-state hypoxic fractions of xenografted DLD-2 human colon carcinomas by administration of basic fibroblast growth factor or suramin.. Br J Cancer.

[OCR_00667] Lin J. C., Song C. W. (1990). Effects of hydralazine on the blood flow in RIF-1 tumors and normal tissues of mice.. Radiat Res.

[OCR_00672] Moulder J. E., Rockwell S. (1984). Hypoxic fractions of solid tumors: experimental techniques, methods of analysis, and a survey of existing data.. Int J Radiat Oncol Biol Phys.

[OCR_00678] Rockwell S., Moulder J. E. (1990). Hypoxic fractions of human tumors xenografted into mice: a review.. Int J Radiat Oncol Biol Phys.

[OCR_00683] Rofstad E. K. (1992). Repopulation between radiation fractions in human melanoma xenografts.. Int J Radiat Oncol Biol Phys.

[OCR_00688] SAPIRSTEIN L. A. (1958). Regional blood flow by fractional distribution of indicators.. Am J Physiol.

[OCR_00692] Vlodavsky I., Fuks Z., Ishai-Michaeli R., Bashkin P., Levi E., Korner G., Bar-Shavit R., Klagsbrun M. (1991). Extracellular matrix-resident basic fibroblast growth factor: implication for the control of angiogenesis.. J Cell Biochem.

